# Microparticles in the blood of patients with systemic lupus erythematosus (SLE): phenotypic characterization and clinical associations

**DOI:** 10.1038/srep36025

**Published:** 2016-10-25

**Authors:** Fariborz Mobarrez, Anna Vikerfors, Johanna T. Gustafsson, Iva Gunnarsson, Agneta Zickert, Anders Larsson, David S. Pisetsky, Håkan Wallén, Elisabet Svenungsson

**Affiliations:** 1 Unit of Rheumatology, Department of Medicine, Solna, Karolinska Institutet, Karolinska University Hospital, SE-171 76 Stockholm, Sweden; 2 Department of Clinical Chemistry and Pharmacology, Akademiska Hospital, SE-751 85 Uppsala, Sweden; 3 Department of Medicine, Duke University Medical Center; Medical Research Service, Durham VA Hospital, NC, USA; 4 Department of Clinical Sciences, Karolinska Institutet, Danderyd Hospital, Division of Cardiovascular Medicine, Stockholm, Sweden

## Abstract

Systemic lupus erythematosus (SLE) is a prototypic autoimmune disease characterized by circulating autoantibodies and the formation of immune complexes. In these responses, the selecting self-antigens likely derive from the remains of dead and dying cells, as well as from disturbances in clearance. During cell death/activation, microparticles (MPs) can be released to the circulation. Previous MP studies in SLE have been limited in size and differ regarding numbers and phenotypes. Therefore, to characterize MPs more completely, we investigated 280 SLE patients and 280 individually matched controls. MPs were measured with flow cytometry and phenotyped according to phosphatidylserine expression (PS^+^/PS^−^), cellular origin and inflammatory markers. MPs, regardless of phenotype, are 2–10 times more abundant in SLE blood compared to controls. PS^−^ MPs predominated in SLE, but not in controls (66% vs. 42%). Selectively in SLE, PS^−^ MPs were more numerous in females and smokers. MP numbers decreased with declining renal function, but no clear association with disease activity was observed. The striking abundance of MPs, especially PS^−^ MPs, suggests a generalized disturbance in SLE. MPs may be regarded as “liquid biopsies” to assess the production and clearance of dead, dying and activated cells, i.e. pivotal events for SLE pathogenesis.

Systemic lupus erythematosus (SLE) is a prototypic autoimmune disease characterized by the production of autoantibodies targeting nuclear and membrane molecules [i.e., antinuclear antibodies (ANA) and antiphospholipid antibodies (aPL)] in association with systemic and local inflammatory manifestations as well as premature vascular disease^1^,^2^,^3^. Lupus primarily affects young women (90%) and, while its etiology is unknown, studies on patients and murine models suggest an important role in pathogenesis for disturbances in cell death as well as the clearance of dead and dying cells. These disturbances may increase the amount of circulating cellular debris which is believed to contain self-antigens (e.g., nucleosomes and DNA) that can trigger B-cells to produce autoantibodies in susceptible individuals^4^,^5^,^6^. While there has been extensive investigation on the molecular properties of the ANAs and aPLs, much less is known about the *in vivo* form of the nuclear antigens. Furthermore, there are few biomarkers that can assess the extent of *in vivo* cell death, tissue localization of dying cells and their clearance.

Among the products of dead and dying cells, microparticles (MPs) provide an important source of bioactive molecules that can induce a broad range of immunological and vascular activities; these particles can also provide a source of nuclear and membrane molecules to drive ANA and aPL production, eventually leading to immune complex formation^7^,^8^. MPs are small membrane-bound vesicles, 0.1 to 1.0 microns in diameter, that can be released from cells undergoing cell activation as well as cell death. A major class of extracellular vesicles (EVs) in the blood, MPs can transmit information from one cell to another and modulate responses through their content of alarmins (e.g., HMGB1), lipid mediators and cytokines as well as nuclear autoantigens; MPs also display tissue factor, which can promote thrombosis^9^,^10^,^11^. MPs are typically identified in terms of size by flow cytometry by light scatter and exposure of phosphatidylserine (PS), an “eat me signal” for the immune system, detected by staining with annexin-V or lactadherin; further characterization involves cell surface markers of the potential cells of origin (e.g., CD41 from platelets)^12^,^13^.

Studies on the expression of MPs in lupus and related disorders have been of limited size and use a variety of analytic techniques for particle assessment. Together, these studies suggest that disturbances in MP expression in SLE are common and extensive although, in some instances, the conclusions of these studies have diverged. Thus, both higher and lower numbers of MPs in the blood of SLE patient compared to those in controls have been reported^14^,^15^. Because of the value of MPs as biomarkers, we have characterized more completely MP expression in SLE through the flow cytometric analysis of blood from large and very well characterized cohort of consecutive SLE patients and compared these profiles to those of individually matched population controls. We further explored if the levels MPs are related to clinical and serological features of SLE.

## Results

### Clinical Characteristics of Patients

Basic clinical characteristics of the patients are presented in Table 1. Median disease duration was 13 years (IQR: 6–23 years). Active disease was considered present if SLAM index (disease activity measurement) was above 6 (49%)^16^.

### Occurrence of Microparticles in Blood

**Figure 1:**
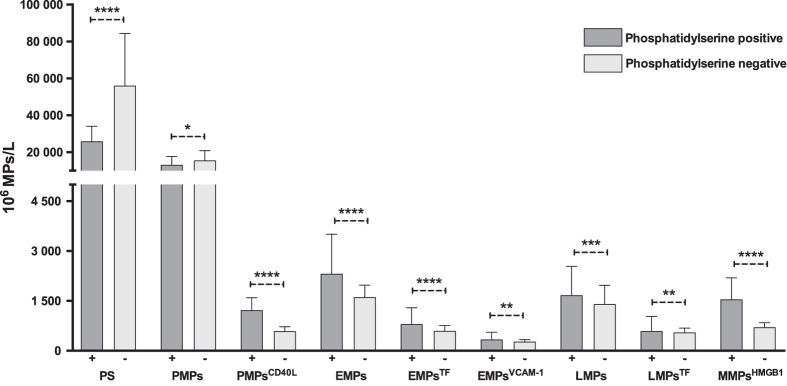
Distribution of phosphatidylserine negative and phosphatidylserine positive microparticles in SLE patients. Microparticles in SLE plasma (n = 280) were detected by flow cytometry and defined as particles less than 1.0 μm in size and either positive or negative for lactadherin-FITC. Each subpopulation was then phenotyped according to origin or expression of inflammation/activation markers. Dark grey = phosphatidylserine positive (PS^+^) microparticles; light grey = phosphatidylserine negative (PS^−^) microparticles; PMPs = platelet microparticles; EMPs = endothelial microparticles; LMPs = leukocyte microparticles; MMPs = monocyte microparticles; CD40L = Cd40 ligand; TF = tissue factor; VCAM-1 = Vascular cell adhesion protein 1; HMGB1 = High mobility group box 1. Each group (PS^+^ vs. PS^−^) was compared with t-test after log transformation of data. Bars represent median values and line indicates interquartile range (25^th^ and 75^th^ percentile). *p < 0.05; **p < 0.01; ***p < 0.001; ****p < 0.0001.

**Figure 2:**
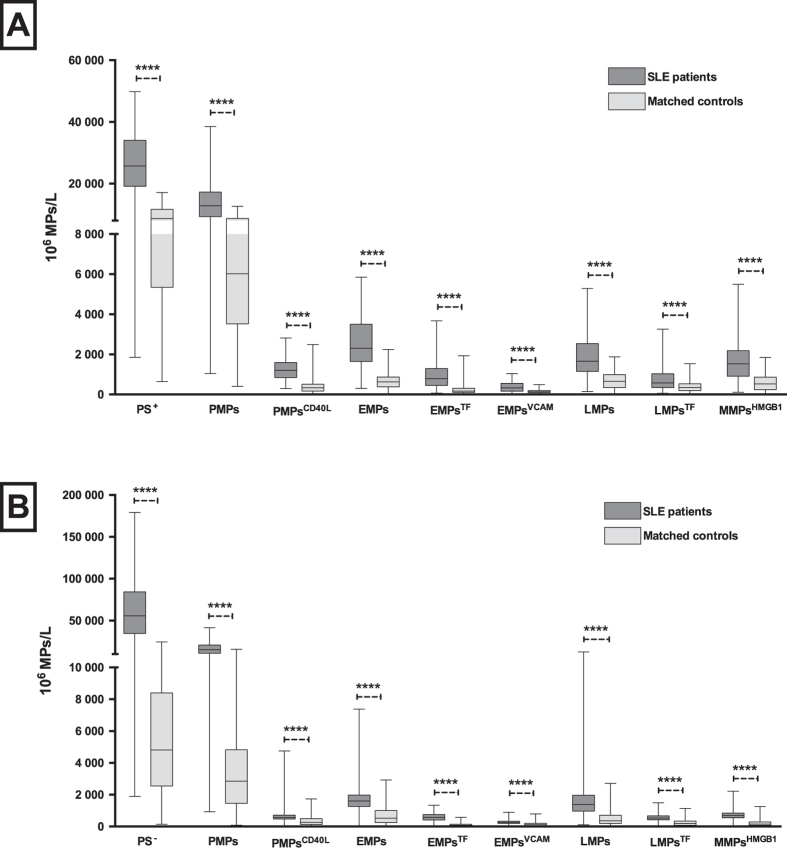
Number and phenotype of lactadherin positive (**A**) or lactadherin negative (**B**) microparticles in SLE patients and matched controls. Microparticles in SLE (n = 280) and matching controls (n = 280) were detected by flow cytometry and defined as particles less than 1.0 μm in size and either positive for lactadherin (**A**) or negative for lactadherin (**B**). Each subpopulation was then phenotyped according to origin or expression of inflammation/activation markers. Dark grey = SLE patients; light grey = matching controls; PMPs = platelet microparticles; EMPs = endothelial microparticles; LMPs = leukocyte microparticles; MMPs = monocyte microparticles; CD40L = Cd40 ligand; TF = tissue factor; VCAM-1 = Vascular cell adhesion protein 1; HMGB1 = High mobility group box 1. Each group (patients vs. controls) was compared with t-test after log transformation of data. Bars represent median values and Platelet microparticles and endothelial microparticles expressing complement *p < 0.05; **p < 0.01; ***p < 0.001; ****p < 0.0001.

Overall, the numbers of MPs measured in the blood were approximately 6 times greater in SLE patients than controls. MPs were initially sub-grouped as PS positive (PS^+^) or PS negative (PS^−^) particles, based on lactadherin binding. In SLE patients, PS^−^ MPs predominated (64%) and were approximately three times more common than PS^+^MPs (Supplementary Figure 1). In contrast, PS^+^ MPs were most common (64%) in controls. Thus, the majority of the MPs measured in the SLE samples are PS^−^ MPs (Fig. 1). We further characterized these MPs in terms of their expression of markers that may indicate a role in inflammation or thrombosis. For this purpose, we assessed the expression of tissue factor, VCAM-1, CD40L and HMGB1 on MPs. By other approaches, levels of these markers have all been shown to be elevated in the blood of SLE patients^17^,^18^,^19^. Of note, previous studies have shown that both sCD40L and HMGB1 can be MP components^20^,^21^. The data presented in Fig. 2 indicate that these markers are expressed on both the PS^−^ and PS^+^ populations.

**Figure 3:**
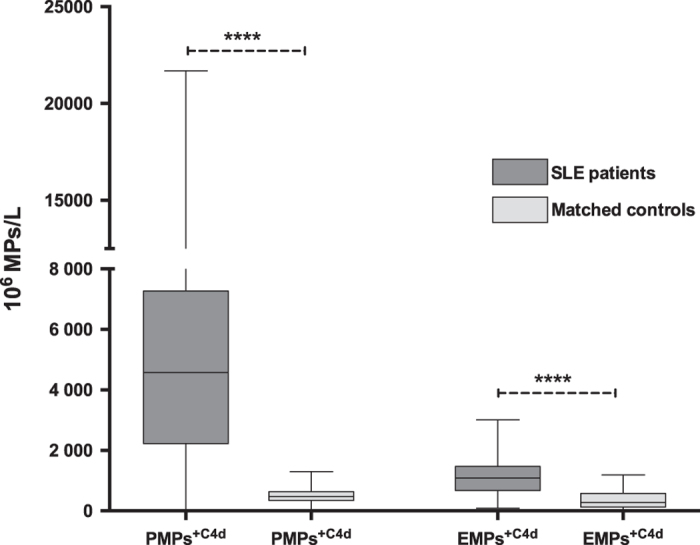
Platelet microparticles and endothelial microparticles expressing complement factor 4d in SLE patients and matched controls. Microparticles in SLE and matching controls were detected by flow cytometry and defined as particles less than 1.0 μm in size and positive for either CD41 (platelet origin) or CD144 (endothelial origin). Later, the expression of complement factor 4d was measured in each subpopulation. PMPs = platelet microparticles; EMPs = endothelial microparticles; C4d = complement factor 4d. Each group (patients vs. controls) was compared with t-test after log transformation of data. Bars represent median values and line indicates interquartile range (25^th^ and 75^th^ percentile). *p < 0.05; **p < 0.01; ***p < 0.001; ****p < 0.0001.

In a further comparison of the levels of the different MP phenotypes with those of individually matched controls, the data indicated that all MP populations, regardless of PS surface marker expression, are more abundant in SLE patients compared to controls (Fig. 2A,B). Platelet, endothelial and leukocyte derived MPs (PMPs, EMPs and LMPs) in blood of patients were approximately 2–7 times higher than those of controls. Activation and inflammation markers, such as CD40L, VCAM-1, TF and HMGB1, expressed on MPs were also much higher in SLE patients. The numbers of C4d-expressing PMPs and EMPs were approximately ten and five times higher, respectively, in SLE patients as compared to controls (Fig. 3). It is of note that measurement of C4d was not performed in the presence of lactadherin, as the reagents for the detection of these proteins are both conjugated with FITC dye. Thus, both PS^+^ and PS^−^ MPs were included when C4d-expressing MPs were counted.

### Associations between microparticles levels and phenotype and SLE features

The levels of PS^−^ and PS^+^ MPs in SLE patients were investigated for associations with clinical and serological variables. In general, levels of PS^−^ MPs showed more convincing associations with SLE features as compared to PS^+^ MPs (significant level p < 0.01 and higher, Table 2 and Supplementary Tables 1 and 2). We chose to consider only associations that remained significant after Bonferroni correction (Table 2); however, in supplementary Table 1 and 2 all significant associations are shown. As these data indicate, levels of PS^−^ MPs were strongly associated with gender (more in females, Table 2) in SLE, while no gender association was observed among controls (Supplementary Tables 3 and 4). Levels of PS^−^ MPs were also associated with measures of renal function as well as with inflammatory markers; TNF-α, TNFR1 and 2, MCP-1 (Table 2). PS^+^ MPs were associated with disease duration (Table 2). Levels of EMPs expressing C4d were positively associated with smoking. Despite these findings, the levels of MPs were not significantly associated with disease activity as assessed by the SLAM^16^, suggesting that increased expression of MPs from many different cell types is common in the course of SLE but may not be directly related to disease activity.

## Discussion

These studies provide new insights into the expression of MPs during SLE and demonstrate that, regardless of PS expression and phenotype, MPs occur in the blood of patients at levels 2 to 10 times greater than those of controls. In our studies, PS^−^ MPs were twice as common than PS^+^ MPs in SLE blood while, in controls, the distribution was reversed. Furthermore, MPs in SLE blood display pro-inflammatory and pro-coagulant molecules (e.g., sCD40L and TF) and can potentially promote the inflammation and thrombosis characteristic of SLE. Together, our results demonstrate widespread disturbances in MP expression among SLE patients and suggest that MP assessments could be valuable assay platforms for studies of cell turnover and development of new biomarkers in SLE.

Our findings build upon previous studies on particle expression in lupus^14^,^22^,^23^,^24^. Thus, Nielsen *et al*. reported that PS^−^ MPs are more frequent in the blood of SLE patients compared to healthy controls, while PS^+^ MPs occurred at similar frequencies in SLE patients and controls^15^. In contrast, our study showed that the levels of both PS^+^ and PS^−^ MPs are increased in SLE blood. The differences in the results of these studies may relate to methodological issues, including the capabilities of different flow cytometers and the use of annexin-V compared to lactadherin to identify PS^12^,^25^. Importantly, we found that the majority of the MPs in SLE blood are PS^−^ MPs, while in controls the majority are PS^+^. Thus, the use of PS expression to define MP numbers may give an incomplete picture of particle expression in SLE.

In our studies, PS^−^MPs in SLE were strongly associated with female gender, similar gender differences were not apparent in controls (Table 2). This is an interesting observation, which should be further investigated as a possible clue to understand the striking female predominance in SLE. The high numbers of PS^−^ MPs could result from masking of PS by other molecules such as β_2_GP1 and annexin-V which are abundant proteins in the circulation^26^,^27^; both of these proteins can bind to PS. Autoantibodies against β_2_GP1 and/or annexin V are frequent in SLE^6^,^28^,^29^; these antibodies could bind to PS^+^ MPs, thus preventing interaction with lactadherin. This possibility is consistent with studies demonstrating that MPs in SLE blood carry increased loads of immunoglobulins on their surface^8^,^24^. Since PS is crucial for the recognition of apoptotic material by phagocytes, increased number of PS^−^ MPs in SLE could persist unrecognized in the circulation, promoting inflammation and thrombosis as well as serving as sources of autoantigens to drive responses or form immune complexes^7^,^30^,^31^.

Platelet MPs were the most abundant particle type both in patients and controls. Furthermore, our data show that PMPs displaying CD40L are more numerous in SLE, regardless of PS expression (Fig. 2A,B). Platelets are the main source of CD40L, producing >90% of soluble CD40L (sCD40L) in blood^32^. As PMPs carry CD40L from the site of platelet activation, they could promote activation of cells in the periphery as well as the immediate surroundings. We have recently demonstrated that assay of CD40L positive MPs may represent a more sensitive approach for sCD40L determinations compared to ELISA assays, suggesting that assay of “soluble’ markers on particles may represent a useful analytic approach^21^. Relevance of such assessment comes from studies indicating that levels of CD40L on immune cells as well as sCD40L are increased in SLE and links to disease activity and activation of B-cells and the type I IFN system have been reported^33^,^34^,^35^.

As our results showed, the total number of EMPs and EMPs expressing VCAM-1 and TF were increased in both PS^+^ and PS^−^ MPs. We have previously reported that circulating VCAM-1 levels can predict cardiovascular events and cardiovascular mortality in SLE^36^,^37^, but the present study is the first to demonstrate increased numbers of EMPs expressing VCAM-1 in SLE. These results are consistent with those other studies indicating ongoing endothelial activation in SLE^38^. The finding of enhanced TF expression on both EMPs and leukocyte MPs, together with previous reports of increased levels of soluble TF in SLE^39^, could indicate an active role of TF positive MPs in thrombosis; functional measurements of particles, however, are needed to evaluate this possibility. In this regard, we previously demonstrated that MPs can promote thrombin formation by both exposing PS (offers a binding site for coagulation factors) and by expressing TF^40^.

In the current study, we detected approximately ten times more C4d^+^ PMPs and five times more C4d^+^ EMPs in patients than in controls. The increased number of MPs expressing C4d in SLE points to ongoing complement activation in lupus. This is also consistent with observations indicating the utility of erythrocyte and platelet-bound C4d levels as a biomarker associated with all-cause mortality, vascular events, and aPL^41^,^42^. The presence of C4d on particles could result from their role as a source of self-antigen to form immune complexes. Related studies have demonstrated the presence of IgG-positive particles in blood of patients as well as the ability of serum antibodies to bind to particles generated *in vitro*^8^,^24^.

The prototype alarmin HMGB1 is a non-histone nuclear protein with diverse immunological activities. Both cell death and immune cell activation can lead to the translocation of HMGB1 from the nucleus into the cytoplasm and the extracellular space. High levels HMGB1 are present in the blood of SLE patients and associated with higher disease activity^43^,^44^,^45^. The data presented herein demonstrate a high frequency of MMPs with HMGB1 in SLE. In a previous study, LPS administration to volunteers induced increased numbers of particles with HMGB1^20^. These results suggest that, during inflammation MPs may be a source of extracellular HMGB1, which can be quantified in blood. As suggested in other studies, the concentration of HMGB1 molecules in proximity with other cytokines on a particle surface could increase immune activity by allowing interaction with multiple receptors simultaneously^46^,^47^.

In assessing clinical association of MP levels in SLE, we found more robust clinical associations with PS^−^ MPs than PS^+^ MPs. In accord with some previous studies^14^,^23^, we did not find convincing associations with disease activity. The lack of a relationship between particle numbers and disease activity could relate to the variety of clinical manifestations assessed by an instrument such as the SLAM and their weighting; furthermore, MPs may play a role in some but not all features of lupus. Since our study was cross-sectional in design, we cannot exclude a stronger association with disease activity in individual patients over time. Nevertheless, the elevation of MPs during putatively inactive disease in some patients may also suggest that current instruments do not adequately reflect disease activity.

While we did not show a relationship of MP levels to disease activity, we did demonstrate that declining renal function is associated with fewer numbers of PS^−^ MPs and PS^−^ PMPs. A recent study demonstrated that galactin-3 binding protein, a component of MPs, can co-localize with immune complexes in renal biopsies; these findings suggest that MPs can be deposited in renal tissue and serve as a source of immune complexes to drive events in lupus nephritis^48^. We also observed strong positive associations between the number of PMPs expressing CD40L and levels of TNFR1 and/or TNFR2. All of which belongs to the tumor necrosis factor receptor superfamily, whose expression may increase during inflammation and apoptosis. TNFRs have also been associated with disease activity in SLE, in particular with active nephritis^49^,^50^. Two recent *in vitro* studies have reported that particles can induce NETosis as well as the production of INF-α and other pro-inflammatory cytokines^38^,^51^. Further studies are needed to understand how particles relate to the different phases of SLE activity.

In conclusion, in this large study, we report striking differences in numbers of various particle types in the blood of SLE patients and matched controls; we also report skewed relative distribution of PS^−^ and PS^+^ MPs. Together, these findings point to widespread disturbances in SLE in the processes generating and clearing MPs and suggest that MP measurement should be explored as a component of “liquid biopsies” to evaluate the ongoing events in the course of SLE.

## Methods

### Study population and clinical data

In this cross-sectional study we included 280 consecutive SLE patients, who received care at the Department of Rheumatology, Karolinska University Hospital 2004–2011. All patients fulfilled at least four of the 1982 revised classification criteria for SLE according to the American College of Rheumatology^3^ were asked to participate. We used no exclusion criteria. 280 “SLE-free” population controls were individually matched to the SLE patients for age, sex and region of living. Controls were identified in the Swedish national population registry, contacted by letter and asked to participate. A rheumatologist interviewed and examined all participants. Medical records were reviewed. Organ manifestations were defined according to criteria^3^. SLE disease activity was assessed with the SLAM^16^ and organ damage with Systemic Lupus International Collaborating Clinics/ACR Damage index (SLICC/ACR-DI)^52^. Myocardial infarction (MI), ischaemic heart disease (IHD, including MI and/or angina), ischaemic stroke, venous thromboembolism (VTE, including venous thromboembolism and/or pulmonary embolism) were verified as previously described^53^. The APS was defined according to Miyakis *et al*., with the exception that not all aPL tests were performed within five years of the clinical event^54^. On the morning of clinical assessment blood samples were drawn into citrated tubes after overnight fasting and centrifuged within 1 hour at 2570 *g* for 20 min in room temperature (RT) and subsequently stored at −80° as platelet-poor plasma (PPP). The local ethic committee at Karolinska University Hospital approved the study and all the experiments were performed in compliance with good clinical practice and good laboratory practice. All study subjects gave written informed consent to participate.

### Laboratory tests including aPL and lupus anticoagulant

High-sensitivity (hs) CRP, fibrinogen, albumin and creatinine were measured with BN ProSpec System (Dade Behring, Deerfield, IL, USA). Complement factor 3 and 4 (C3 and C4) were analyzed using IMMAGE^TM^ system (Beckman Coulter, Brea, CA, USA). Cystatin C was analyzed on Architect Ci8200 analyzer with cystatin C reagents from Gentian (Moss, Norway). Levels of TNFR1 (DY225), TNFR2 (DY726), VCAM (DY809), IP10 (DY266), and MCP-1 (DY279) were analyzed with commercial sandwich ELISA kits (R&D Systems, Minneapolis, MN, USA). Estimated (e) glomerular filtration rate (GFR) was calculated from serum cystatin C (eGFRcystatin C) results in mg/L as previously described^55^. Other variables were determined by routine clinical tests. Antinuclear antibodies (ANA) were analysed by indirect immunofluorescence (IFL) on HEp-2 cells (Immunoconcepts, Sacramento, CA, USA). Antibodies to specific nuclear antigens (dsDNA, SSA-Ro52, SSA-Ro60, SSB, Sm) and phospholipids (cardiolipin IgG, IgM, IgA and β_2_-glycoprotein1 IgG, IgM, IgA) were analysed by multiplexed bead technology (Luminex) using BioPlex 2200 system (Bio-Rad, Hercules, CA, USA) according to the specifications of the manufacturer. The cut-off for anti-cardiolipin (aCL) and anti-β_2_-glycoprotein1 (aβ_2_GP1) fulfills the 99^th^ percentile as described^54^. LA was determined using a modified Dilute Russell Viper Venom method (Biopool, Umea, Sweden) using Bioclot lupus anticoagulant. In February 2010, a new test procedure using two integrated tests (one dRRVT and one APTT) became routine. Less than 5% of the samples were analyzed according to this procedure.

### Measurement of MPs

PPP was thawed in a water bath for approximately 5 minutes (37 °C) and then centrifuged at 2000*g* for 20 min at RT, in order to further remove any debris or cells that may interfere with the analysis. The supernatant was then re-centrifuged at 13000*g* for 2 min at RT. Subsequently, 20 μL of the supernatant (which contains the MPs) were incubated for 20 minutes in the dark with phalloidin-Alexa 660 (cell-fragment marker, Invitrogen, Paisley, UK)^56^, lactadherin-FITC (binds to PS, Haematologic Technologies, VT, USA) and either CD41-PE (platelet MP; PMPs, abcam, Cambridge, UK), CD45-PC7 (leukocyte MP; LMPs, Beckman Coulter, Brea, CA, USA) or CD144-APC (endothelial MPs; EMPs, AH diagnostics, Stockholm, Sweden). In addition, exposure of CD142-PE (tissue factor; TF, BD, NJ, USA) was measured on EMPs and LMPs, CD40 Ligand (L) (CD154; BD, San Jose, CA, USA) on PMPs, vascular cell adhesion molecule 1 (VCAM-1; CD106-PE, AH diagnostics, Stockholm, Sweden) on EMPs, high-mobility group protein B1 (HMGB1; R&D Systems, Minneapolis, USA) on monocyte MPs (MMPs, CD14-PC7, Beckman Coulter, Brea, CA, USA) and C4d (anti-C4d-FITC, ALPCO Diagnostics, Salem, NH, USA) on PMPs and EMPs. MPs were measured by flow cytometry on a Beckman Gallios instrument (Beckman Coulter, Brea, CA, USA). The MP gate was determined using Megamix beads (0.5–3.0 μm, BioCytex, Marseille) (Fig. 2 in supplementary). MPs were defined as particles less than 1.0 μm in size (as MPs are between 0.1–1.0 μm in diameter) and positive or negative to lactadherin and cell markers as described above, i.e. two populations of MPs based on PS expression. Conjugate isotype-matched immunoglobulins with no reactivity against human antigens were used as negative controls. In the present study, results are shown as numbers of MPs (MP counted x standard beads added/L)/standard beads counted (FlowCount, Beckman Coulter, CA, USA). The intra- and interassay coefficients of variation for MP measurement were less than 9% respectively.

### Statistical analysis

Prior to statistical analysis, data were log transformed, if necessary, to obtain a normal distribution. Patients and matched controls were compared with paired tests; McNemar´s test or paired t-test depending on data type. Within the patient and control group, associations between MPs levels and clinical/laboratory characteristics were investigated with linear regression or t-test, and results are reported as p-value and direction of association. The Bonferroni correction was used to adjust for multiple comparisons (Table 2 and Supplementary Tables 1–4). Statistical analysis was performed using JMP software (SAS Institute, v12.0, Cary, North Carolina, USA).

## Additional Information

**How to cite this article**: Mobarrez, F. *et al*. Microparticles in the blood of patients with systemic lupus erythematosus (SLE): phenotypic characterization and clinical associations.. *Sci. Rep.* **6**, 36025; doi: 10.1038/srep36025 (2016).

## Supplementary Material

Supplementary Information

## Figures and Tables

**Table 1:** Basic clinical characteristics of SLE patients and controls.

	SLE	Controls	*p*
Number	280	280	*ns*
Age (mean ± SD)	47.4 ± 14.7	47.6 ± 14.7	*ns*
Gender Female (n, %)	258 (92%)	259 (92%)	*ns*
Disease duration, years (median [25:75])	13 [6:23]	—	—
Smoking, Current (n, %)	57 (20%)	37 (13%)	<*0.05*
**Medication** (at inclusion, if not otherwise stated)			
Prednisolone (n, %) Mean dose: 8.6 mg	161 (58%)	2 (1%)	<*0.0001*
Azathioprine	54 (19%)	—	—
Hydroxychloroquine	93 (33%)	—	—
Mycophenolate mofetil	17 (6%)	—	—
Rituximab (ever)	24 (9%)	—	—
Warfarin	43 (15%)	1 (1%)	<*0.0001*
Low dose Acetylsalicylic acid	51 (18%)	9 (3%)	<*0.0001*
Statins	34 (12%)	12 (4%)	<*0.0001*
**Lupus Manifestations**			
Butterfly (n, %)	147 (53%)	0 (0%)	—
Discoid (n, %)	56 (20%)	0 (0%)	—
Photosensitivity (n, %)	197 (70%)	51 (18%)	<*0.0001*
Oral ulcers (n, %)	97 (35%)	8 (3%)	<*0.0001*
Arthritis (n, %)	235 (84%)	10 (4%)	<*0.0001*
Pleuritis (n, %)	98 (35%)	2 (1%)	<*0.0001*
Pericarditis (n, %)	48 (17%)	0 (0%)	—
Serositis (n, %)	110 (39%)	2 (1%)	<*0.0001*
Nephritis (n, %)	110 (39%)	1 (1%)	<*0.0001*
Psychosis (n, %)	7 (3%)	2 (1%)	*ns*
Seizures (n, %)	30 (11%)	5 (2%)	<*0.0001*
Leucopenia (n, %)	138 (49%)	3 (1%)	<*0.0001*
Lymphopenia (n, %)	146 (52%)	2 (1%)	<*0.0001*
Thrombocytopenia (n, %)	61 (22%)	2 (1%)	<*0.0001*
Hemolytic anemia (n, %)	16 (6%)	1 (1%)	<*0.001*
aDNA, ever (n, %)	193 (69%)	—	—
aSm, ever (n, %)	42 (15%)	—	—
Immunological criteria (n, %)	200 (71%)	—	—
SLAM > 6 (n, %)	137 (49%)	—	—
**Autoantibodies at Inclusion**			
dsDNA (n, %)	101 (36%)	3 (1%)	<*0.0001*
Nucleosomes (n, %)	122 (44%)	1 (0%)	<*0.0001*
Sm (n, %)	52 (19%)	2 (0%)	<*0.0001*
RNP68 (n, %)	25 (9%)	0 (0%)	<*0.0001*
SSA (n, %)	123 (44%)	4 (1%)	<*0.0001*
SSB (n, %)	64 (23%)	9 (3%)	<*0.0001*
Lupus anticoagulant (n, %)	63 (23%)	—	—
Cardiolipin IgG (n, %)	53 (19%)	—	—
Cardiolipin IgM (n, %)	23 (8%)	—	—
Cardiolipin IgA (n, %)	45 (16%)	—	—
β_2_GP-1 IgG (n, %)	56 (20%)	—	—
β_2_GP-1 IgM (n, %)	26 (9%)	—	—
β_2_GP-1 IgA (n, %)	44 (16%)	—	—
**Inflammation Markers**			
hs CRP (mg/L) (median [25:75])	1.5 [0.6:5.0]	0.9 [0.5:2.2]	<*0.0001*
C3 (g/L) (median [25:75])	0.86 [0.7:1.01]	—	—
C4 (g/L) (median [25:75])	0.14 [0.1:0.19]	—	—
Fibrinogen (g/L) (median [25:75])	3.9 [3.2:4.8]	3.8 [3.2:4.4]	*ns*
Creatinine (μmol/L) (median [25:75])	69 [59:84]	66 [59:74]	<*0.0001*
Cystatin C GFR (mL/min/1,73 m^2^) (median [25:75])	76 [55:101]	108 [93:121]	<*0.0001*
IL-6 (ng/L) (median [25:75])	3.3 [2.2:6.3]	—	—
TNFα (pg/L) (median [25:75])	1.8 [1.0:3.0]	—	—
TNFR1 (pg/L) (median [25:75])	1697 [1279:2478]	1306 [1087:1564]	<*0.0001*
TNFR2 (pg/L) (median [25:75])	5200 [3851:8055]	3600 [2875:4300]	<*0.0001*
IP-10 (pg/L) (median [25:75])	199 [121:379]	72 [50:101]	<*0.0001*
MCP-1 (pg/L) (median [25:75])	184 [112:292]	72 [29:112]	<*0.0001*
**Other Parameters**			
APS (n, %)	47 (17%)	0 (0%)	—
Any arterial event (n, %)	35 (13%)	3 (1%)	<*0.001*
Any vascular event (n, %)	68 (24%)	7 (2%)	<*0.001*
Venous thromboembolism (VTE) (n, %)	41 (15%)	4 (1%)	<*0.001*

Distributions are given as % or median (interquartile range). Patients and matched controls were compared with paired tests; McNemar’s test or paired t-test depending on data type. aDNA = anti-DNA antibody; aSM = Anti-Smith antibodies; SLAM = Systemic Lupus Activity Measure^16^; dsDNA = doublestranded DNA; ; Sm = Smith; RNP = ribonucleoprotein; SSA = Sjogrens syndrome antigen A; SSB = Sjogrens syndrome antigen B; β_2_GP-1 = beta_2_glykoprotein-1; hsCRP = high sensitivity C reactive protein; C3/C4 = Complement factor 3/4; GFR = GFR - glomerular filtration rate; IL-6 = interleukin 6; TNF = Tumor necrosis factor; TNFR = Tumor necrosis factor receptor; IP10 = Interferon gamma-induced protein 10; MCP-1 = Monocyte Chemoattractant Protein-1. APS = Antiphospholipid syndrome, according to Miyakis *et al*.^53^.

**Table 2:** Associations between Microparticles and SLE features in 280 SLE patients.

	PS negative MPs	PS positive MPs
Gender Female (n = 280)	[+] Total PS negative MPs (***) [+] PMPs (*)	
Smoking current (n = 280)	[+] EMPs^C4d^ (*)	[+] EMPs^VCAM1^ (*)
Inflammation Markers		
Cystatin C GFR (n = 271)	[+] Total PS negative MPs (***) [+] PMPs (*)	
TNF-a (n = 207)	[−] Total PS negative MPs (*)	
TNFR1 (n = 275)	[−] Total PS negative MPs (**) [−] PMPs (*)	
TNFR2 (n = 276)	[−] Total PS negative MPs (*)	
MCP-1 (n = 275)	[+] LMPs (*)	

Values are given as direction [+/−] and MP phenotype followed by asterisk: ***p < 0.001, **p < 0.01, *p < 0.0.5. p-values (t-test) represent adjustment according to Bonferroni test. Blank = non significant (p-value > 0.05). GFR = glomerular filtration rate; TNF = Tumor necrosis factor; TNFR = Tumor necrosis factor receptor; MCP-1 = Monocyte Chemoattractant Protein-1.
