# Supplementary material for: Microparticles in the blood of patients with systemic lupus erythematosus (SLE): phenotypic characterization and clinical associations
Source: Sci Rep. 2016 Oct 25;6:36025. doi: 10.1038/srep36025 (PMC5078765; doi:10.1038/srep36025)
Supplement: Supplementary Information [file srep36025-s1.pdf]

Fariborz Mobarrez*†, Anna Vikerfors†, Johanna T Gustafsson, Iva Gunnarsson, Agneta Zickert, Anders Larsson, David S Pisetsky, Håkan Wallén and Elisabet Svenungsson.

Supplementary Figure 1. Amount and distribution of phosphatidylserine negative (PS^-) and phosphatidylserine positive (PS^+) MPs in 280 SLE patients and 280 controls. Circles are proportional to the total number of MPs among SLE patients and controls. SLE patients had roughly 6 times more total MPs (i.e. PS^+ + PS^- MPs) than controls; 3 times more PS^+ MPs and 10 times more PS^- MPs.

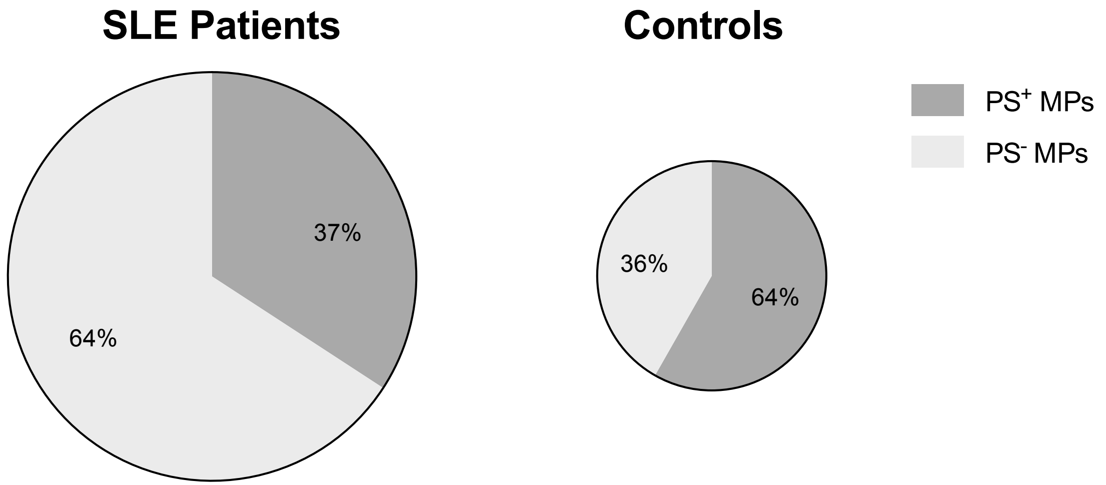

Circles are proportional to the total number of MPs among SLE patients and controls. SLE patients had roughly 6 times more total MPs (i.e. PS^+ + PS^- MPs) than controls; 3 times more PS^+ MPs and 10 times more PS^- MPs.

Fariborz Mobarrez*†, Anna Vikerfors†, Johanna T Gustafsson, Iva Gunnarsson, Agneta Zickert, Anders Larsson, David S Pisetsky, Håkan Wallén and Elisabet Svenungsson.

Supplementary Figure 2. Flow cytometric analysis of microparticles.
The microparticle (MP) gate was determined using Megamix beads (0.5, 0.9 and 3.0 µm beads) (A). Conjugate isotype-matched immunoglobulins with no reactivity against human antigens were used as negative controls (B). MPs were defined as particles less than 1.0 µm in size (the MP-gate in panel A) and positive or negative to lactadherin (C), i.e. PS$^-$ and PS$^+$ MPs. Each subpopulation was further phenotyped according to origin and expression of inflammation and activation markers.

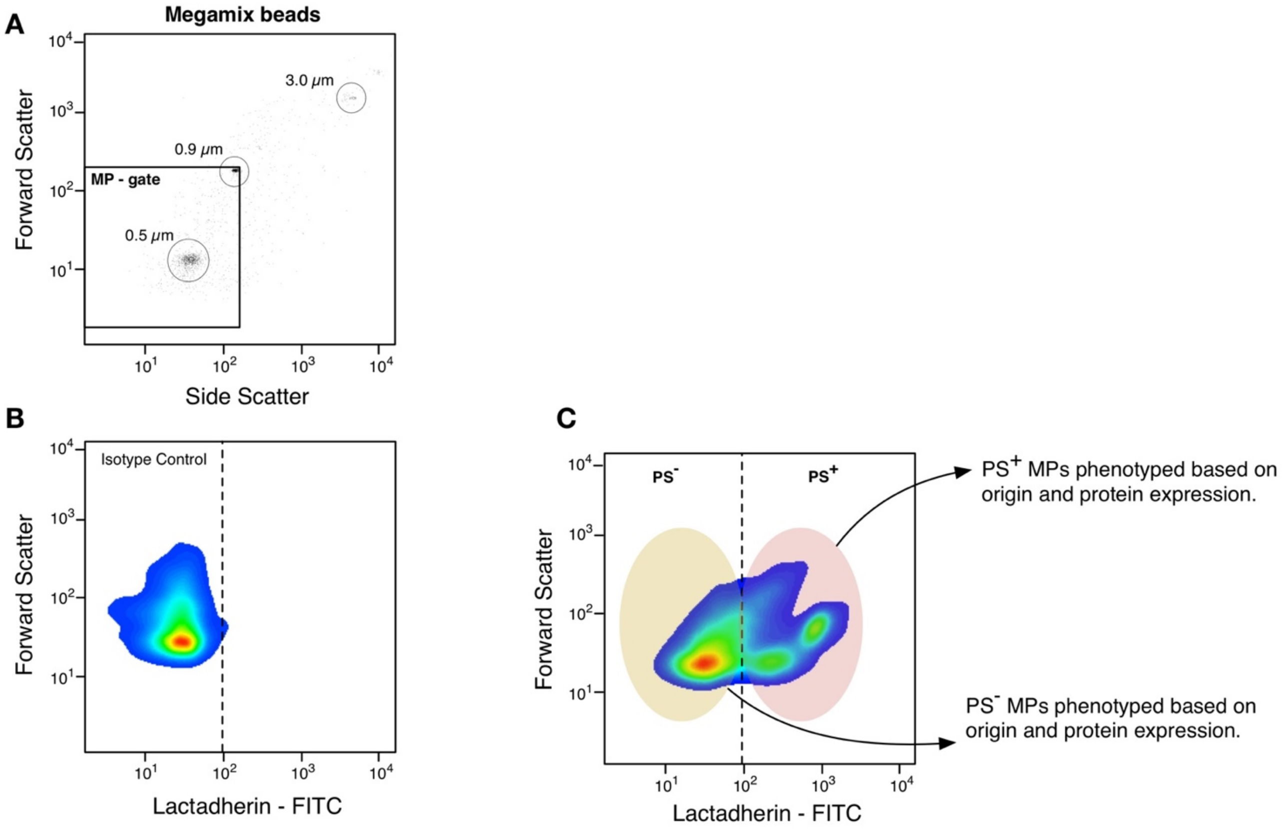

Supplementary Table 1. Demographics, SLE criteria and their relationship to phosphatidylserine negative MPs in 280 SLE patients.

	PS^- MPs	PMPs	PMPs^{CD40L}	EMPs	EMPs^{VCAM}	EMPs^{TF}	LMPs	LMPs^{TF}	MMPs^{HMGB1}	PMPs^{C4d}	EMPs^{C4d}
Age (n=280)			(-) <0.05								
Gender Female (n=280)	(+) <0.0001 ***	(+) <0.001 *									
Disease duration (n=280)	(+) <0.01									(+) <0.05	
Smoking Current (n=280)	(+) <0.01	(+) <0.01									(+) <0.001*
**LUPUS MANIFESTATIONS**											
Butterfly (n=280)		(+) <0.05								(+) <0.05	
Discoid (n=280)											
Photosensitivity (n=280)											
Oral ulcers (n=280)											
Arthritis (n=280)								(-) <0.01			
Pleuritis (n=280)				(+) <0.05			(+) <0.0001 **		(+) <0.05		
Pericarditis (n=280)											
Serositis (n=280)				(+) <0.05			(+) <0.0001 *		(+) <0.05	(+) <0.05	
Nephritis (n=280)	(-) <0.05			(-) <0.05							
Psychosis (n=280)											
Seizures (n=280)											
Leucopenia (n=280)											
Lymphopenia (n=280)							(-) <0.05				
Thrombocytopenia (n=280)							(+) <0.05				
Hemolytic anemia (n=280)											
aDNA, ever (n=280)		(+) <0.05	(+) <0.05				(+) <0.01				
aSm, ever (n=280)	(-) <0.05										
Immunological criteria (n=280)		(+) <0.05	(+) <0.05			(-) <0.05	(+) <0.05				
SLAM >6 (n=277)											
**AUTOANTIBODIES AT INCLUSION**											
dsDNA (n=279)		(+) <0.01									
Nucleosomes (n=280)		(+) <0.05					(-) <0.01				
Sm (n=280)	(-) <0.05										
RNP68 (n=280)											
SSA (n=280)											
SSB (n=280)				(+) <0.01			(+) <0.05				

Lupus anticoagulant (n=280)		(-) <0.05									
Cardiolipin IgG (n=262)				(+) <0.05		(+) <0.05		(+) <0.05			
Cardiolipin IgM (n=262)					(+) <0.05			(+) <0.05	(+) <0.05		
Cardiolipin IgA (n=262)				(+) <0.05		(+) <0.05		(+) <0.05			
β_2GP-1 IgG (n=262)				(+) <0.05		(+) <0.05	(+) <0.05	(+) <0.05			
β_2GP-1 IgM (n=262)				(+) <0.05	(+) <0.05	(+) <0.05					
β_2GP-1 IgA (n=262)				(+) <0.05		(+) <0.05		(+) <0.05			(+) <0.05
**INFLAMMATION**											
hs CRP (n=274)	(-) <0.05	(-) <0.05									
C3 (n=264)	(-) <0.05	(-) <0.001				(+) <0.05	(-) <0.01	(+) <0.05			
C4 (n=264)		(-) <0.05				(+) <0.05	(-) <0.001	(+) <0.05			
Fibrinogen (n=271)	(-) <0.05	(-) <0.05				(+) <0.001					
Creatinine (n=277)	(-) <0.05	(-) <0.05	(+) <0.05								
Cystatin C GFR (n=271)	(+) <0.0001 ***	(+) <0.001 *	(-) <0.01								
IL-6 (n=207)											
TNF-α (n=207)	(-) <0.001 *	(-) <0.05									
TNFR1 (n=275)	(-) <0.001 **	(-) <0.001 *	(+) <0.05								
TNFR2 (n=276)	(-) <0.001 *	(-) <0.05	(+) <0.01					(-) <0.05			
IP-10 (n=274)							(+) <0.01	(-) <0.01	(-) <0.05		
MCP-1 (n=275)							(+) <0.0001 ***	(+) <0.05			
**OTHER PARAMETERS**											
APS (n=280)											
Any arterial event (n=280)				(+) <0.05							
Any vascular event (n=280)					(-) <0.05						
VTE (n=280)											

*Values are given as direction (+/-) and p-value of association (t-test) for nominal/ordinal data. Blank = non significant (p-value>0.05). The p-values that were still significant after adjustment according to Bonferroni are labeled with asterisk: *** p<0.001, ** p<0.01, * p<0.05. aDNA = anti-DNA antibody; aSM = Anti-Smith antibodies; SLAM = Systemic Lupus Activity Measure [16]; dsDNA = doublestranded DNA; ; Sm = Smith; RNP = ribonucleoprotein; SSA = Sjogrens syndrome antigen A; SSB = Sjogrens syndrome antigen B; β_2GP-1 = beta$_2$glykoprotein-1; hsCRP = high sensitivity C reactive protein; C3/C4 = Complement factor 3/4; GFR = GFR - glomerular filtration rate; IL-6 = interleukin 6; TNF = Tumor necrosis factor; TNFR = Tumor necrosis factor receptor; IP10 = Interferon gamma-induced protein 10; MCP-1 = Monocyte Chemoattractant Protein-1; APS = Antiphospholipid syndrome, according to Miyakis et al [53]; VTE = Venous thromboembolism.*

Supplementary Table 2. Demographics, SLE criteria and their relationship to phosphatidylserine positive MPs in 280 SLE patients.

	PS^{+} MPs	PMPs	$PMPs^{CD40L}$	EMPs	$EMPs^{VCAM}$	$EMPs^{TF}$	LMPs	$LMPs^{TF}$	$MMPs^{HMGB1}$	$PMPs^{C4d}$	$EMPs^{C4d}$
Age (n=280)	(-) <0.01				(+) <0.05						
Gender Female (n=280)											
Disease duration (n=280)	(-) <0.05			(+) <0.05	(+) <0.001 *					(+) <0.05	
Smoking Current (n=280)				(-) <0.05			(-) <0.05				(+) <0.001*
**LUPUS MANIFESTATIONS**											
Butterfly (n=280)										(+) <0.05	
Discoid (n=280)											
Photosensitivity (n=280)							(-) <0.05				
Oral ulcers (n=280)											
Arthritis (n=280)			(+) <0.05								
Pleuritis (n=280)								(-) <0.05			
Pericarditis (n=280)											
Serositis (n=280)								(-) <0.05		(+) <0.05	
Nephritis (n=280)			(-) <0.05								
Psychosis (n=280)	(+) <0.05										
Seizures (n=280)								(-) <0.05			
Leucopenia (n=280)											
Lymphopenia (n=280)			(+) <0.05				(+) <0.05				
Thrombocytopenia (n=280)											
Hemolytic anemia (n=280)											
aDNA, ever (n=280)											
aSm, ever (n=280)									(+) <0.05		
Immunological criteria (n=280)											
SLAM >6 (n=277)											
**AUTOANTIBODIES AT INCLUSION**											
dsDNA (n=279)	(-) <0.05		(+) <0.05								
Nucleosomes (n=280)											
Sm (n=280)											
RNP68 (n=280)											
SSA (n=280)											
SSB (n=280)											
Lupus anticoagulant (n=280)											
Cardiolipin IgG (n=262)				(-) <0.05					(-) <0.05		
Cardiolipin IgM (n=262)				(-) <0.01							(+) <0.05

Cardiolipin IgA (n=262)								
β_2GP-1 IgG (n=262)								
β_2GP-1 IgM (n=262)								
β_2GP-1 IgA (n=262)		(+) <0.05			(-) <0.05		(-) <0.01	
**INFLAMMATION MARKERS**		(-) <0.05						
hs CRP (n=274)								
C3 (n=264)		(+) <0.05						(+) <0.05
C4 (n=264)		(+) <0.05						
Fibrinogen (n=271)								
Creatinine (n=277)								
Cystatin C GFR (n=271)		(+) <0.05						
IL-6 (n=207)								
TNF-α (n=207)								
TNFR1 (n=275)								
TNFR2 (n=276)								
IP-10 (n=274)		(+) <0.05						
MCP-1 (n=275)				(-) <0.05				
**OTHER PARAMETERS**	(-) <0.05							
APS (n=280)			(+) <0.05			(-) <0.05		
Any arterial event (n=280)			(-) <0.05					
Any vascular event (n=280)				(+) <0.01				
VTE (n=280)								

*Values are given as direction (+/-) and p-value of association (t-test) for nominal/ordinal data. Blank = non significant (p-value>0.05). The p-values that were still significant after adjustment according to Bonferroni are labeled with asterisk: *** p<0.001, ** p<0.01, * p<0.05. aDNA = anti-DNA antibody; aSM = Anti-Smith antibodies; SLAM = Systemic Lupus Activity Measure [16]; dsDNA = doublestranded DNA; ; Sm = Smith; RNP = ribonucleoprotein; SSA = Sjogrens syndrome antigen A; SSB = Sjogrens syndrome antigen B; β_2GP-1 = beta$_2$glykoprotein-1; hsCRP = high sensitivity C reactive protein; C3/C4 = Complement factor 3/4; GFR = GFR - glomerular filtration rate; IL-6 = interleukin 6; TNF = Tumor necrosis factor; TNFR = Tumor necrosis factor receptor; IP10 = Interferon gamma-induced protein 10; MCP-1 = Monocyte Chemoattractant Protein-1; APS = Antiphospholipid syndrome, according to Miyakis et al [53]; VTE = Venous thromboembolis*

Supplementary Table 3. Demographics and their relationship to phosphatidylserine negative microparticles in 280 controls

	PS^{-} MPs	PMP	PMP$^{CD40 L}$	EMP	EMP^{VCAM}	EMP^{TF}	LMP	LMP^{TF}	MMP^{HMGB1}	PMP ^{C4d}	EMP^{C4d}
Age (n=280)					(-) 0.05						
Gender Female (n=280)											(+) 0.05
Smoking Current (n=280)	(+) 0.05	(+) 0.05			(+) 0.05						
**INFLAMMATION MARKERS**											
hs CRP (n=279)											
Fibrinogen (n=266)									(+) 0.05	(+) 0.05	
Creatinine (n=275)									(+) 0.05	(+) 0.05	
Cystatin C GFR (n=264)									(-) 0.05	(-) 0.05	
TNFR1 (n=274)									(+) 0.05		
TNFR2 (n=274)											
IP-10 (n=271)	(+) 0.05	(+) 0.05				(+) 0.05			(+) 0.05		
MCP-1 (n=271)											

*Values are given as direction (+/-) and p-value of association (t-test) for nominal/ordinal data. Blank = non significant (p-value>0.05). The p-values that were still significant after adjustment according to Bonferroni are labeled with asterisk: *** p<0.001, ** p<0.01, * p<0.05. hsCRP = high sensitivity C reactive protein; GFR = GFR - glomerular filtration rate; IL-6 = interleukin 6; TNF = Tumor necrosis factor; TNFR = Tumor necrosis factor receptor; IP10 = Interferon gamma-induced protein 10; MCP-1 = Monocyte Chemoattractant Protein-1.*

Fariborz Mobarrez*†, Anna Vikerfors†, Johanna T Gustafsson, Iva Gunnarsson, Agneta Zickert, Anders Larsson, David S Pisetsky, Håkan Wallén and Elisabet Svenungsson.

Supplementary Table 4. Demographics and their relationship to phosphatidylserine positive microparticles in 280 controls

	PS+ MPs	PMPs	PMPs$^{CD40 L}$	EMPs	EMPs^{VCAM}	EMPs^{TF}	LMPs	LMP^{TF}	MMPs^{HMGB1}	PMPs ^{C4d}	EMPs^{C4d}
Age (n=280)											
Gender Female (n=280)				(+) 0.05							(+) 0.05
Smoking Current (n=280)		(+) 0.05					(+) 0.05		(+) 0.05		
**INFLAMMATION MARKERS**											
hs CRP (n=279)											
Fibrinogen (n=266)	(+) 0.05		(+) 0.05 *				(+) 0.05 *		(+) 0.05	(+) 0.05	
Creatinine (n=275)				(+) 0.05						(+) 0.05	
Cystatin C GFR (n=264)						(-) 0.05				(-) 0.05	
TNFR1 (n=274)				(+) 0.05 *		(+) 0.05					
TNFR2 (n=274)									(+) 0.05		
IP-10 (n=271)		(+) 0.05		(+) 0.05							
MCP-1 (n=271)			(+) 0.05		(+) 0.05		(+) 0.001 **	(+)0.001 **	(+) 0.001 **		

*Values are given as direction (+/-) and p-value of association (t-test) for nominal/ordinal data. Blank = non significant (p-value>0.05). The p-values that were still significant after adjustment according to Bonferroni are labeled with asterisk: *** p<0.001, ** p<0.01, * p<0.05. hsCRP = high sensitivity C reactive protein; GFR = GFR - glomerular filtration rate; IL-6 = interleukin 6; TNF = Tumor necrosis factor; TNFR = Tumor necrosis factor receptor; IP10 = Interferon gamma-induced protein 10; MCP-1 = Monocyte Chemoattractant Protein-1.*
